# Sigma-1 Receptor as a Novel Therapeutic Target in Diabetic Kidney Disease

**DOI:** 10.3390/ijms252413327

**Published:** 2024-12-12

**Authors:** Dora B. Balogh, Judit Hodrea, Adar Saeed, Marcell Cserhalmi, Alexandra Rozsahegyi, Tamas Lakat, Lilla Lenart, Attila J. Szabo, Laszlo J. Wagner, Andrea Fekete

**Affiliations:** 1 MTA-SE Lendület “Momentum” Diabetes Research Group, 1083 Budapest, Hungary; balogh.dora.bianka@semmelweis.hu (D.B.B.); hodrea.judit@semmelweis.hu (J.H.); adaramedi992@gmail.com (A.S.); cserhalmi.marcell@semmelweis.hu (M.C.); szandi7777@gmail.com (A.R.);; 2 Pediatric Center, MTA Center of Excellence, Semmelweis University, 1083 Budapest, Hungary; szabo.attila@semmelweis.hu; 3 Department of Surgery, Transplantation, and Gastroenterology, Semmelweis University, 1082 Budapest, Hungary; laszlo.drwagner@gmail.com

**Keywords:** diabetic kidney disease, Sigma-1 receptor, fibrosis

## Abstract

Diabetic kidney disease (DKD) is the leading cause of chronic kidney disease. Current treatments for DKD do not halt renal injury progression, highlighting an urgent need for therapies targeting key disease mechanisms. Our previous studies demonstrated that activating the Sigma-1 receptor (S1R) with fluvoxamine (FLU) protects against acute kidney injury by inhibiting inflammation and ameliorating the effect of hypoxia. Based on these, we hypothesized that FLU might exert a similar protective effect in DKD. Diabetes was induced in male Wistar rats using streptozotocin, followed by a seven-week FLU treatment. Metabolic and renal parameters were assessed along with a histological analysis of glomerular damage and fibrosis. The effects of FLU on inflammation, hypoxia, and fibrosis were tested in human proximal tubular cells and normal rat kidney fibroblasts. FLU improved renal function and reduced glomerular damage and tubulointerstitial fibrosis. It also mitigated inflammation by reducing *TLR4*, *IL6*, and *NFKB1* expressions and moderated the cellular response to tubular hypoxia. Additionally, FLU suppressed TGF-β1-induced fibrotic processes and fibroblast transformation. These findings suggest that S1R activation can slow DKD progression and protect renal function by modulating critical inflammatory, hypoxic, and fibrotic pathways; therefore, it might serve as a promising novel drug target for preventing DKD.

## 1. Introduction

Chronic kidney disease (CKD) is a significant global health issue, impacting over 840 million individuals worldwide, with diabetic kidney disease (DKD) being the most common cause [1]. DKD develops in approximately 30–40% of diabetic patients and is the primary cause of end-stage renal disease (ESRD) in adults. It is associated with poor quality of life, high morbidity, mortality, and economic costs [2,3]. The rising global prevalence of diabetes mellitus (DM) is reflected in the growing incidence of DKD, further exacerbating healthcare challenges.

Until recently, the prevention and management of DKD primarily relied on glycemic control, blood pressure management, and renin–angiotensin system inhibitors (RAASis) treatment. Although RAASis reduce albuminuria and slow the decline in glomerular filtration rate (GFR), their effectiveness in preventing ESRD remains modest [4]. The novel class of glucose-lowering agents, the sodium–glucose cotransporter 2 inhibitors (SGLT2is), undoubtedly changed the therapeutic landscape; they reduce the risk of major adverse cardiovascular events and eGFR decline in DKD [5]. Consequently, the most recent KDIGO guidelines recommend using SGLT2is for patients with type 2 DM, CKD, or an eGFR > 20 mL/min per 1.73 m^2^ [6]. However, particularly in people with type 1 DM (T1DM), where SGLT2is are not allowed, the risk of DKD progression still persists, highlighting the unmet need for additional treatment strategies [7].

The pathophysiology of DKD is multifaceted. Prolonged hyperglycemia causes metabolic disturbances, leading to glomerular hemodynamic changes, albuminuria, and progressive renal damage at the glomerular, tubular, and tubulointerstitial levels. Chronic inflammation and tubular hypoxia are the early events contributing to glomerular hyperfiltration, podocyte injury, endothelial dysfunction, and tubulointerstitial fibrosis, the final common pathway of all these processes [8,9,10]. As current therapeutic options are limited in targeting these pathways, novel solutions are needed that directly mitigate fibrosis and reduce renal complications.

The Sigma-1 receptor (S1R) is a highly conserved chaperone protein primarily studied in the central nervous system, but it is also expressed in peripheral tissues [11,12]. S1R modulates several cellular processes, including calcium signaling, inflammation, oxidative stress, and apoptosis. Our previous research identified S1R as a novel mediator of renoprotective mechanisms in ischemia–reperfusion injury. Fluvoxamine (FLU), a selective serotonin reuptake inhibitor with a high affinity for S1R, significantly improved survival and renal function, reduced tubular damage, and decreased inflammation in a rat model of acute kidney injury (AKI) induced by sublethal renal ischemia [13]. Furthermore, we demonstrated that perfusion of the donor organ with FLU effectively reduced cold and warm ischemic injury in a rat kidney transplantation model [14]. These findings offer a rationale for exploring the therapeutic potential of S1R activation in treating DKD.

Furthermore, we also revealed that FLU-mediated S1R activation has an antifibrotic effect in vitro. It prevents cytoskeletal rearrangement and reduces extracellular matrix (ECM) accumulation in trabecular meshwork cells after exposure to profibrotic stimuli [15,16].

Building on these previous results, we designed this study to explore the potential renoprotective benefits of S1R activation in T1DM-related DKD. We hypothesized that the S1R agonist FLU improves kidney function, reduces structural damage, and mitigates renal fibrosis in a streptozotocin-induced diabetic rat model. To investigate its effects on inflammatory, hypoxic, and fibrotic pathways, we conducted a series of in vitro experiments.

## 2. Results

### 2.1. FLU Treatment Did Not Affect T1DM-Induced Metabolic Alterations

The classical metabolic parameters were evaluated after seven weeks of T1DM induction to assess disease progression. Diabetic rats exhibited impaired weight gain and elevated blood glucose, triglycerides, and transaminase levels, consistent with T1DM pathology. FLU treatment did not affect these parameters (Table 1).

### 2.2. FLU Slowed the Progression of Renal Functional Impairment

T1DM rats showed significant renal impairment, as indicated by elevated serum creatinine, BUN, and protein excretion levels and a marked reduction in creatinine clearance, confirming the development of DKD. Both serum creatinine and BUN levels were reduced in the FLU-treated group compared to untreated diabetic rats. Although protein excretion and creatinine clearance also showed improvements following FLU treatment, these changes did not reach statistical significance (Table 2).

### 2.3. FLU Administration Alleviated Renal Tubular Damage

**Figure 1:**
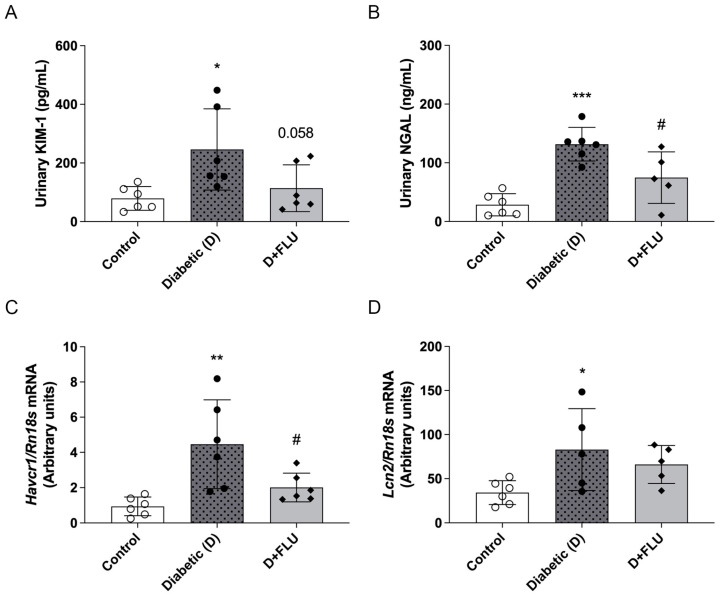
Fluvoxamine mitigated tubular damage. Control, diabetic (D), and fluvoxamine-treated diabetic (D + FLU) rats. (**A**,**B**) Urinary concentrations of kidney injury molecule-1 (KIM-1) and neutrophil gelatinase-associated lipocalin (NGAL). (**C**,**D**) KIM-1 (*Havcr1*) and NGAL (*Lcn2*) mRNA expressions. mRNA expressions were normalized to *Rn18S*. Values are represented as means ± SD. * *p* < 0.05 vs. Control, ** *p* < 0.01 vs. Control, *** *p* < 0.001 vs. Control, # *p* < 0.05 vs. D (*n* = 6/group).

Measuring kidney injury molecule-1 (KIM-1) and neutrophil gelatinase-associated lipocalin (NGAL) is crucial for early kidney injury detection and monitoring, as they are sensitive biomarkers that reflect tubular damage and the progression of renal dysfunction [17,18]. Diabetic rats showed elevated urinary concentrations and renal mRNA expression levels of KIM-1 and NGAL, confirming T1DM-induced renal tubular injury. FLU treatment effectively reduced urinary KIM-1 and NGAL concentrations and decreased renal *Havcr1* (KIM-1) expression (Figure 1A–D).

### 2.4. Mesangial Matrix Expansion Was Ameliorated by FLU Treatment

**Figure 2:**
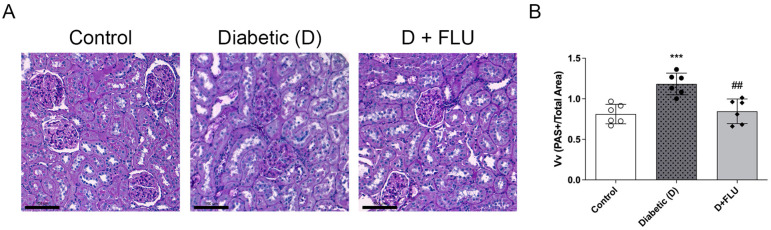
Fluvoxamine mitigated mesangial matrix expansion in diabetic kidneys. (**A**) Representative PAS-stained kidney sections of control, diabetic (D), and fluvoxamine-treated diabetic (D + FLU) rats. (**B**) Mesangial fractional volume values (Vv) were defined by the ratio of the PAS-stained mesangial area to the glomerular tuft area. Original magnification = 200×. Scale bar = 100 µm. Values are represented as means ± SD. *** *p* < 0.001 vs. Control, ## *p* < 0.01 vs. D (*n* = 6/group).

Histological changes corresponded with the observed decline in renal function. Control rat kidneys displayed well-organized glomeruli that were compactly enclosed within Bowman’s capsule. In contrast, diabetic rats exhibited disorganized glomerular structures characterized by hypertrophy, basal membrane thickening, and mesangial matrix expansion (Figure 2A). FLU treatment effectively reduced DM-induced mesangial matrix expansion, as indicated by decreased PAS-positive glomerular areas (Figure 2B).

### 2.5. FLU Treatment Prevented Tubulointerstitial Fibrosis

**Figure 3:**
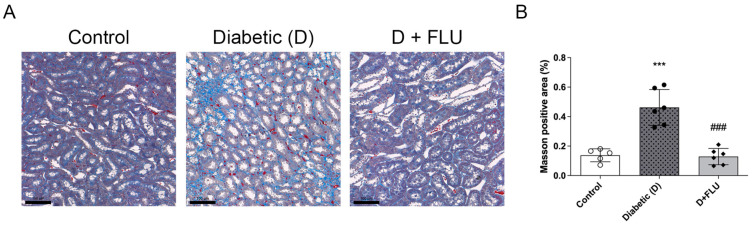
Fluvoxamine diminished tubulointerstitial fibrosis. (**A**) Representative Masson’s trichrome-stained kidney sections of control, diabetic (D), and fluvoxamine-treated diabetic (D + FLU) rats. (**B**) Renal tubulointerstitial fibrosis was quantified by the ratio of Masson-positive (blue), glomerulus-free areas (px) to the total area in the kidney cortex. Original magnification = 200×. Scale bar = 100 µm. Values are represented as means ± SD. *** *p* < 0.001 vs. Control, ### *p* < 0.001 vs. D (*n* = 6/group).

Tubulointerstitial fibrosis, a hallmark of DKD, was assessed by Masson’s trichrome staining. Compared to controls, diabetic rats showed massive interstitial fibrosis and tubular atrophy with a narrow lumen and thickened tubular basement membrane (Figure 3A). FLU significantly mitigated fibrotic changes, highlighting its potential to prevent renal fibrosis (Figure 3B).

### 2.6. LPS-Induced Inflammation Was Reduced by FLU Treatment

**Figure 4:**
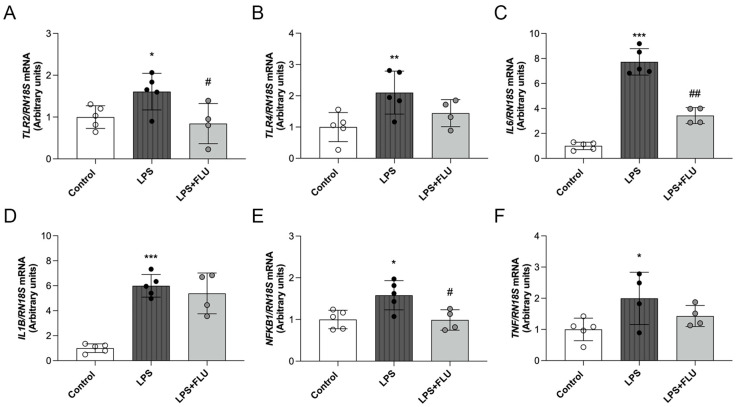
Fluvoxamine reduced inflammatory responses in HK-2 cells. Control, lipopolysaccharide (LPS), and LPS + fluvoxamine-treated (LPS + FLU) HK-2 cells. (**A**–**F**) toll-like receptor-2 (*TLR2*), toll-like receptor-4 (*TLR4*), interleukin-6 (*IL6*), interleukin-1β (*IL1B*), nuclear factor κB (*NFKB1*), and tumor necrosis factor-α (*TNF*) mRNA expression. mRNA expressions were normalized to *RN18S*. Values are represented as means ± SD. * *p* < 0.05 vs. Control, ** *p* < 0.01 vs. Control, *** *p* < 0.001 vs. Control, # *p* < 0.05 vs. LPS, ## *p* < 0.01 vs. LPS (*n* = 4–5/group).

Chronic inflammation plays a crucial role in the progression of DKD. The interconnected pathway of toll-like receptors (TLRs), NF-κB, and interleukins is a central component of the inflammatory response. To assess whether S1R activation can mitigate tubular inflammation, we examined the mRNA expression of *TLR2*, *TLR4 NFKB1*, *IL1B*, *IL6*, and *TNF* after LPS induction in HK-2 cells. LPS treatment markedly increased *TLR2* and *TLR4* expression (Figure 4A,B), leading to a subsequent rise in *NFKB1* and proinflammatory cytokine levels (Figure 4C–F). FLU treatment reduced the LPS-induced increase in *TLR2*, *NFKB1*, and *IL6* expressions.

### 2.7. FLU Ameliorated the Cellular Response to Tubular Hypoxia

**Figure 5:**
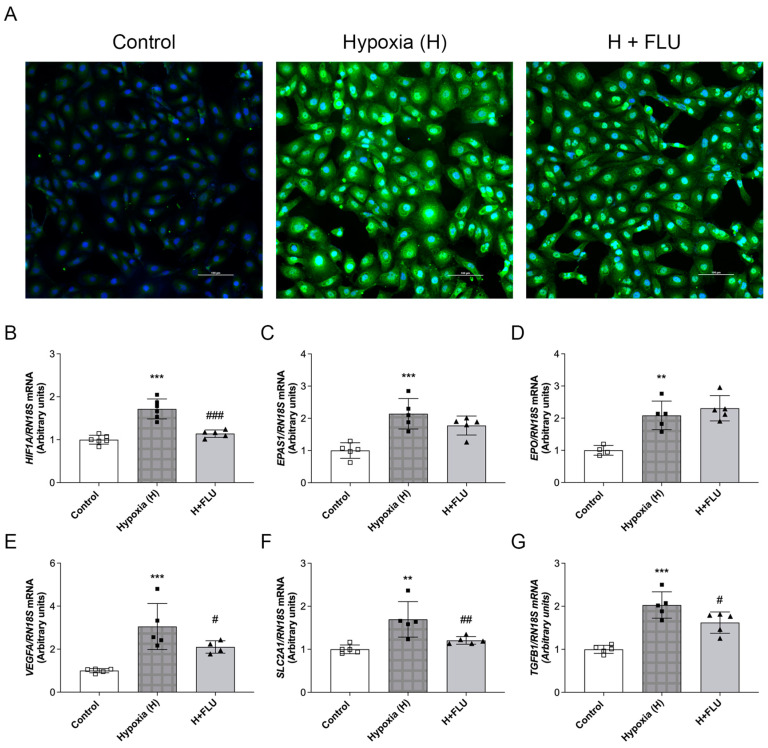
Fluvoxamine abolished the activation of the HIF-1α pathway and decreased pro-fibrotic response. Control, hypoxia (H), and hypoxia + fluvoxamine-treated (H + FLU) HK-2 cells. (**A**) Representative immunocytochemistry staining of HIF-1α (green: HIF-1α; blue: nucleus; original magnification 200×. Scale bar = 100 μm). (**B**–**G**) mRNA expression of hypoxia-inducible factor-1α (*HIF1A*), hypoxia-inducible factor-2α (*EPAS1*), erythropoietin (*EPO*), vascular endothelial growth factor A (*VEGFA*), glucose transporter-1 (*SLC2A1*), and transforming growth factor-β1 (*TGFB1*) normalized to *RN18S*. Values are represented as means ± SD. ** *p* < 0.01 vs. Control, *** *p* < 0.001 vs. Control, # *p* < 0.05 vs. Hypoxia, ## *p* < 0.01 vs. Hypoxia, ### *p* < 0.001 vs. Hypoxia (*n* = 4–5/group).

To investigate the effects of FLU on tubular hypoxia, HK-2 cells were exposed to 1% O_2_ for 2 h to mimic hypoxic conditions. HIF proteins play a crucial role in the cellular response to hypoxia, regulating genes involved in oxygen homeostasis, angiogenesis, and metabolic adaptation. Under hypoxic conditions, HK-2 cells showed significant activation of the hypoxia response pathway. Immunofluorescence analyses revealed elevated HIF-1α protein levels (Figure 5A), accompanied by increased mRNA expression of both *HIF1A* and *EPAS1* (encoding HIF-2α) (Figure 5B,C). Downstream markers of hypoxic stress, including *EPO*, *VEGFA*, and *SLC2A1*, were also upregulated (Figure 5D–F). FLU suspended HIF-1α protein level and *HIF1A* mRNA expression elevation, indicating a milder hypoxic injury. Moreover, FLU treatment also decreased the mRNA expression of *VEGFA* and *SLC2A1*, while *EPAS1* and *EPO* levels remained unaffected.

Hypoxia is a crucial driver of renal fibrosis. Our findings demonstrated increased mRNA expression of *TGFB1* under hypoxic conditions that were effectively moderated by FLU (Figure 5G).

### 2.8. FLU Suppressed TGF-β1-Induced Fibrosis and Fibroblast Transformation in NRK-49F Cells

**Figure 6:**
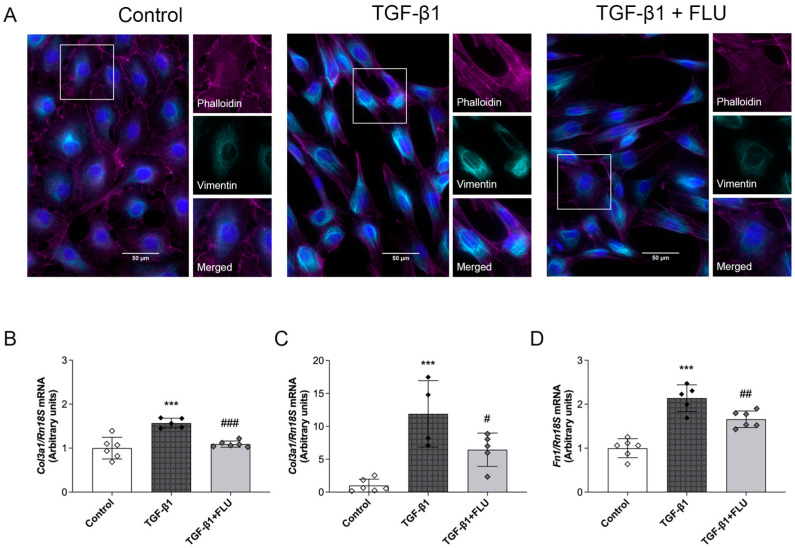
Fluvoxamine mitigated ECM deposition and cellular morphology changes in NRK-49F cells. Control, transforming growth factor-β1 (TGF-β1), and TGF-β1 + fluvoxamine-treated (TGF-β1 + FLU) NRK-49F cells. (**A**) Representative immunocytochemistry staining of phalloidin and vimentin (magenta: phalloidin; cyan: vimentin; blue: nucleus. Original magnification 600×. Scale bar = 50 μm). (**B**–**D**) collagen type I α1 (*Col1a1*), collagen type III α1 (*Col3a1*), fibronectin (*Fn*) mRNA expression. mRNA expressions were normalized to Rn18S. Values are represented as means ± SD. *** *p* < 0.001 vs. Control, # *p* < 0.05 vs. TGF-β1, ## *p* < 0.01 vs. TGF-β1, ### *p* < 0.001 vs. TGF-β1 (*n* = 4–6/group).

TGF-β1-treated NRK-49F renal fibroblast cells were used to investigate the potential antifibrotic effect of the FLU. TGF-β1 induced the reorganization of the actin cytoskeleton, which was demonstrated by the significant actin accumulation and the formation of dense clumps and stress fibers on phalloidin-stained NRK-49F cells. FLU treatment reduced the formation of stress fibers and actin clumps, suggesting the role of S1R activation in mitigating cytoskeletal rearrangement. Vimentin, a type III intermediate filament, a key marker of epithelial–mesenchymal transition (EMT), was also assessed. FLU significantly reduced TGF-β1-induced vimentin accumulation and the formation of long intermediate filaments (Figure 6A).

*Col1a1*, *Col3a1*, and *Fn* mRNA expression were upregulated upon TGF-β1 induction. FLU treatment effectively suppressed the gene expression of these fibrosis-related ECM components, suggesting its antifibrotic potential (Figure 6B–D).

## 3. Discussion

The authors’ current treatments of DM that primarily focus on glycemic management, blood pressure regulation, and hemodynamic changes have limited efficacy in halting the progression of kidney damage, particularly in patients with T1DM. As multifactorial processes beyond metabolic disturbances drive DKD, we targeted a novel, non-metabolic pathway as a potential therapeutic approach. We focused on modulating the S1R, a highly conserved chaperone protein primarily studied in the central nervous system regulating key cellular processes, including inflammation and hypoxia. As an inter-organelle signaling molecule, S1R is also present in peripheral tissues. In particular, our research group has identified the specific renal localization of this receptor, highlighting its potential in different kidney diseases. Our promising preclinical results suggest that S1R activation provides renoprotective effects in acute models. Additionally, S1R shows antifibrotic potential in extrarenal organs, including the eyes and lungs. Based on these findings, we extended our studies to investigate its effects on DKD [13,14,15,16]. We assessed the effect of S1R agonist FLU through in vivo and in vitro models, particularly focusing on the critical pathological pathways of DKD to determine its potential as an adjunct therapy for preserving kidney function.

Long-term FLU treatment improved renal function and reduced serum creatinine and BUN levels in the STZ-induced T1DM rats, demonstrating its ability to protect renal function. Similar findings have been reported with the S1R agonist PRE-084, which improved renal function in non-diabetic CKD rat models [19,20]. Our previous study revealed that pretreatment with dehydroepiandrosterone (DHEA), an endogenous S1R agonist, improves survival and renal function after sublethal renal ischemia-reperfusion injury [13]. Additionally, DHEA has been shown to protect against pressure overload-induced kidney injury in ovariectomized rats [21]. These findings further underscore the renoprotective potential of S1R activation across various forms of kidney injury.

Both experimental and clinical studies have demonstrated that renal tubular damage is a crucial contributor to the progression of DKD [22]. KIM-1 and NGAL are sensitive biomarkers of early tubular damage, often revealing kidney injury before more overt clinical signs emerge [23]. Recent research has demonstrated that KIM-1 and NGAL levels are reliable predictors of ESRD and eGFR decline, independent of other factors, even in non-proteinuric T1DM patients [18,24,25]. Our study showed elevated levels of KIM-1 and NGAL in diabetic rats, consistent with our previous work identifying these markers as early indicators of tubular damage in T1DM [26]. FLU treatment reduced urinary KIM-1 and NGAL levels and the renal *Havcr1* (KIM-1) expression, further supporting its protective role in maintaining tubular integrity.

The progression of DKD is associated with structural alterations, with glomerular basement membrane thickening being one of the main histological features observed in T1DM patients. In our study, diabetic rats exhibited excessive glomerular basement membrane thickening and mesangial matrix expansion. FLU treatment attenuated these alterations, suggesting that S1R activation not only preserves kidney function but also plays a crucial role in preventing structural damage.

Renal fibrosis represents the final common pathway driving the progression of chronic kidney disease [7]. In our study, Masson’s trichrome staining demonstrated a marked reduction in interstitial fibrosis and tubular atrophy in FLU-treated diabetic rats, which further underscores the potential of FLU to slow or even halt the progression of DKD toward ESRD. In line with our results, the S1R agonist PRE-084 reduced ECM deposition in a rat model of adenine-induced renal fibrosis, further supporting the therapeutic relevance of targeting S1R [19].

The progression of DKD is closely linked to systemic and local inflammation, which accelerates tubulointerstitial damage and fibrosis. TLRs are also found in non-immune cells such as kidney tubular epithelial cells, endothelial cells, podocytes, and mesangial cells [27]. In patients with DKD, TLR2 and TLR4 play a significant role in driving the inflammatory processes involved in disease pathogenesis [28]. Moreover, TLR4 activation promotes podocyte injury and interstitial fibrosis in STZ-induced diabetic nephropathy in mice [29]. Based on these observations, we designed an in vitro model to investigate the anti-inflammatory potential of FLU. Our experiments demonstrated that FLU remarkably attenuated LPS-induced inflammation in tubular epithelial cells. The reduction in *TLR2* expression underscores the role of S1R activation in regulating immune responses.

The NF-κB pathway acts as a bridge between TLR2 activation and the inflammatory response. Once TLR2 is activated, it initiates a signaling cascade that activates NF-κB, resulting in the transcription of proinflammatory genes that exacerbate renal inflammation. To further investigate LPS-induced NF-κB activation, we measured the mRNA expression of inflammatory mediators. LPS treatment markedly increased *NFKB*, *IL1B*, *IL6*, and *TNF*, while FLU significantly halted NF-κB downstream signaling. These results align with previous studies showing that FLU moderates inflammatory responses in human whole blood cell cultures by decreasing LPS-induced IL-6 and IL-1β production [30]. Similarly, PRE-084 has been shown to reduce *TNFA* and *IL1B* mRNA expression in LPS-activated murine microglial BV-2 cells [31]. Consistent with these findings, DHEA pretreatment has also been found to alter IL-1β and IL-6 gene expression in a model of acute renal failure [32]. Our data and the existing literature demonstrate that S1R activation regulates inflammatory responses in various tissues and cells, highlighting the therapeutic potential of S1R-targeting drugs for modulating inflammatory pathways.

Tubular hypoxia is a crucial driver of DKD progression. As early as 1994, Körner et al. demonstrated increased O_2_ consumption in proximal tubules isolated from STZ-treated diabetic rats compared to controls, a finding confirmed by numerous studies [33]. Palm et al. reported reduced renal tissue O_2_ tension in diabetic rats using Clark-type microelectrodes, and later studies using MRI further validated heightened tubular O_2_ consumption in diabetic kidneys [34,35]. HIF-1α is a crucial regulator of cellular adaptation to hypoxia. To explore whether FLU provides renoprotection through the modulation of tubular hypoxia, we applied an in vitro model of low-grade hypoxia. As expected, HIF-1α expression was upregulated, consistent with our previous findings [26]. This elevation was significantly reduced by FLU treatment. Key downstream hypoxia-related markers, such as *VEGFA* and *SLC2A1*, were also diminished. These results suggest that S1R activation mitigates hypoxic injury in the proximal tubules.

Hypoxia triggers several pathways contributing to EMT, primarily through HIF-1α. In various cell types, HIF-1α has been shown to drive EMT via a TGF-β1-dependent pathway [36]. To better understand the TGF-β1 and hypoxia signaling pathways, as they could be a treatment target for fibrosis, we measured TGF-β1 levels under hypoxic conditions. FLU reduced hypoxia-induced *TGFB1* expression, suggesting that its antifibrotic effects may be partly mediated by modulating the hypoxic response. While a detailed investigation of the impact of FLU on hypoxia-induced molecular pathways was beyond the scope of this investigation, our findings underscore its potential in mitigating hypoxia-related kidney damage.

Tubulointerstitial fibrosis results from excess connective tissue accumulation during reparative or reactive processes. While collagen I is the most abundant matrix protein in renal fibrosis, other types, including collagens III, V, VI, VII, and XV, as well as the adhesive glycoprotein fibronectin, also accumulate. Fibroblasts are considered the primary matrix-secreting cells, which are highly responsive to TGF-β1, the critical regulator of myofibroblast differentiation in fibrosis [37]. Therefore, we investigated the antifibrotic potential of FLU using NRK-49F renal fibroblasts treated with TGF-β1. Our in vitro findings revealed that FLU suppressed TGF-β1-induced fibroblast transformation and ECM deposition. By inhibiting the expression of *Col1a1*, *Col3a1*, and *Fn*, FLU demonstrated its ability to reduce fibrosis at the cellular level. In line with our findings, FLU has been shown to decrease the *COL1A1* mRNA expression in TGF-β1-stimulated NIH/3T3 fibroblasts [38]. Furthermore, FLU attenuated fibroblast activation and proliferation in neonatal rat cardiac fibroblasts. In this model, pretreatment with FLU before TGF-β1 stimulation led to a marked decrease in the expression of key fibrosis markers [39]. These findings suggest that FLU effectively modulates fibrotic pathways across different cell types and tissues, highlighting its potential as a therapeutic strategy for mitigating renal fibrosis.

Our findings provide data for the therapeutic potential of S1R activation in DKD, demonstrating its multifaceted protective effects on renal function, inflammation, hypoxia, and fibrosis. These results underscore the promise of S1R-targeting strategies as novel adjunct therapies to prevent DKD and improve outcomes in kidney fibrosis, particularly in T1DM, where the risk of progression remains high despite current treatment options.

## 4. Materials and Methods

### 4.1. Study Approval

Animal procedures were conducted according to the regulations of the Committee on the Care and Use of Laboratory Animals of the Council of Animal Care at Semmelweis University, Budapest, Hungary (PEI/001/380-4/2013).

### 4.2. Materials

Chemicals and reagents were purchased from Sigma-Aldrich (St. Louis, MO, USA), and all standard plastic laboratory equipment was obtained from Sarstedt (Nümbrecht, Germany) unless stated otherwise.

### 4.3. Experimental Design of In Vivo Experiments

Experiments were conducted on eight-week-old male Wistar rats (*Rattus norvegicus*) acquired from Toxi-Coop Toxicological Research Center (Dunakeszi, Hungary) and kept in groups of three per cage under controlled light and temperature conditions (12:12 h light–dark cycle at 24 ± 2 °C). Animals were provided ad libitum access to a standard rodent diet and tap water. T1DM was induced with a single intraperitoneal injection of 65 mg/bwkg streptozotocin (STZ) dissolved in 0.1 M citrate buffer (pH 4.5) following an overnight fast. Successful induction of T1DM was confirmed by measuring blood glucose levels from the tail vein three times using a D-Cont Ideal device (77 Elektronika, Budapest, Hungary). Rats with postprandial peripheral blood glucose values exceeding 15 mmol/L 72 h after STZ injection were considered diabetic and enrolled in the study.

After the onset of T1DM, diabetic animals were randomly divided into two groups (*n* = 6/group) and treated for seven weeks by oral gavage either with isotonic saline (NaCl 154 mmol/L) as vehicle (D) or fluvoxamine–maleate (20 mg/bwkg/day, D + FLU). Age-matched Wistar rats receiving an equivalent volume of a single dose of citrate buffer without STZ and daily saline served as non-diabetic controls. Following the seven-week treatment, the rats were anesthetized using a mixture of 75 mg/bwkg ketamine (Richter Gedeon, Budapest, Hungary) and 10 mg/bwkg xylazine (Medicus Partner, Biatorbagy, Hungary). Terminal blood samples were drawn from the abdominal aorta to euthanize the animals. Blood, urine, and tissue samples were collected and stored at −80 °C to analyze metabolic parameters, assess renal function (creatinine, blood urea nitrogen, and protein excretion), and perform molecular biology measurements.

### 4.4. Measurement of Metabolic and Renal Parameters

Serum and urinary parameters were analyzed photometrically with a Hitachi 912 chemistry analyzer (Roche Hitachi, Basel, Switzerland). Creatinine clearance and protein excretion were assessed from urine samples collected over 24 h. Urinary levels of KIM-1 and NGAL were quantified with rat-specific enzyme-linked immunosorbent assay (ELISA) (R&D Systems, Minneapolis, MN, USA).

### 4.5. Renal Histology

Renal cortical and cortico-medullary regions were isolated under a light microscope, fixed in 4% buffered formalin, and embedded in paraffin. Sections were cut at a thickness of 5 µm and imaged using a Zeiss AxioImager A1 light microscope (Zeiss, Jena, Germany). The sections were stained with PAS to evaluate mesangial matrix expansion. Twenty glomerular fields at 400× magnification were randomly selected for each animal, excluding incomplete glomeruli along the sample edges. The mesangial area (pixels containing purple-stained mesangial matrix) was quantified as a ratio relative to each glomerulus’s glomerular tuft area (total pixels within the glomerulus).

Masson’s trichrome staining was performed to assess tubulointerstitial fibrosis. For each animal, sections were stained, and ten fields at 200× magnification were randomly selected from the cortical and cortico-medullary regions. The extent of fibrosis was quantified as the ratio of the Masson-stained fibrotic area (pixels containing stained interstitial fibrotic tissue) to the total area (total pixels within the field) in each selected field.

Regions of interest were selected using Panoramic Viewer software version 1.15.2 (3DHISTECH, Budapest, Hungary). Analysis was performed double-blindly using computer-assisted morphometry with Adobe Photoshop CS6 (Adobe Inc., San José, CA, USA) and ImageJ software (https://imagej.net/ij/, accessed on 9 December 2024, U.S. National Institutes of Health, Bethesda, MD, USA).

### 4.6. Experimental Design of In Vitro Experiments

Human proximal tubular epithelial cells (HK-2; LGC Standards, cat. no. CRL-2190, American Type Culture Collection, Manassas, VA, USA) and normal rat kidney fibroblast cells (NRK-49F; LGC Standards, cat. no. CRL-1570, American Type Culture Collection) were cultured in Dulbecco’s modified Eagle’s medium (DMEM; Gibco, Thermo Fisher Scientific, Waltham, MA, USA) supplemented with 10% fetal bovine serum (FBS; Gibco) and 1% penicillin/streptomycin (Gibco). Cells were incubated at 37 °C in a humidified atmosphere containing 5% CO_2_ and 95% air. Depending on the experiments, cells were plated in either 24-well plates or 0.1% gelatin-coated 8-well tissue culture chambers (Biologix, Saint Louis, MO, USA). For reverse transcription-quantitative polymerase chain reaction (RT-qPCR), HK-2 cells were plated at 10^5^ cells/well and NRK-49F cells at 1.8 × 10^4^ cells/well. For immunocytochemistry (ICC), HK-2 cells were plated at 6 × 10^4^ cells/well and NRK-49F cells at 7 × 10^4^ cells/well. Before treatment, a 24 h or 4 h growth arrest period in a serum-free medium was implemented across all experiments for HK-2 and NRK-49F cells, respectively. For all experiments, untreated cells were used as controls.

Three different models were applied in parallel to investigate the effect of FLU on hypoxic, inflammatory, and fibrotic pathways. In the *hypoxia model*, after 24 h of pretreatment with 10 µM FLU or vehicle, HK-2 cells were placed in a bold line stage top CO_2_/O_2_ incubator (Okolab, Ottaviano, Italy), which was flushed with a gas mixture of 1% O_2_, 5% CO_2_, and 94% N_2_, and then incubated for 2 h at 37 °C. To evaluate the effect of FLU on the *inflammatory response*, HK-2 cells were incubated for 24 h with 1 µg/mL of LPS, either alone or in combination with 10 µM FLU. To assess the *anti-fibrotic effect* of FLU, NRK-49F cells were used and treated with 10 ng/mL of TGF-β1 (R&D Systems, Minneapolis, MN, USA), either alone or in combination with 10 µM FLU, for 48 h.

### 4.7. Immunocytochemistry

Cells were washed twice with PBS, fixed in 4% paraformaldehyde, re-washed, and then permeabilized with 0.1% Triton X-100 for 10 min. After repeated washing, samples were blocked with 1% BSA–10% goat serum PBS solution with 0.1% Tween 20 for 1 h at room temperature (RT). HK-2 cells were incubated with anti-HIF-1α primary antibody (Abcam, Cambridge, UK 1:100, overnight, 4 °C) followed by secondary antibody Alexa Fluor 488 chicken anti-rabbit (Invitrogen, Carlsbad, CA, USA; 1:500, 1 h, RT). NRK-49F cells were incubated with anti-vimentin (Cell Signaling Technology, Danvers, MA, USA 1:500, 2 h, RT) and then with Alexa Fluor 488 goat anti-mouse secondary antibody (Invitrogen, 1 h, RT). F-actin was immunostained by incubation with Alexa 546-phalloidin (Invitrogen, 1:40, 1 h, RT). Nuclei were counterstained with Hoechst 33342 (Invitrogen, 1 µg/mL) for 10 min. Samples were visualized under a Nikon Eclipse Ti2 microscope (Nikon Instruments, Melville, NY, USA).

### 4.8. Quantitative Reverse Transcription Polymerase Chain Reaction (RT-qPCR)

Total RNA was isolated using the RT050 Total RNA Isolation Mini Kit (Geneaid Biotech, New Taipei City, Taiwan). The quality and quantity of the RNA were measured on a NanoDrop ND-1000 spectrophotometer (Baylor College of Medicine, Houston, TX, USA). Complementary DNA (cDNA) (500 ng each of *EPAS1*, *EPO*, *GLUT1*, *HIF1A*, *SLC2A1*, and *TGFB1*; 1000 ng of *IL1B*, *IL6*, *NFKB1*, *TLR2*, *TLR4*, and *TNF* mRNA from HK2-2 cells; and 650 ng of *Col1a1*, *Col3a1*, *Fn1*, *Havcr1*, and *Lcn2* mRNAs from NRK-49F cells) was reverse-transcribed using a First Strand cDNA Synthesis Kit for RT-PCR (Thermo Fisher Scientific). Amplification was performed in a reaction mixture containing 1 μL of cDNA, 10 μL of SYBR Green I Master Mix (Roche Diagnostics, Mannheim, Germany), and 10 pmol/μL of each specific primer (Invitrogen). Data analysis was conducted using LightCycler 480 software version 1.5.0 (Roche Diagnostics). Gene expression levels were normalized to 18S ribosomal RNA (*Rn18s* or *RN18S*) expression within the same samples (Table 3).

### 4.9. Statistical Analysis

Data are presented as means ± standard deviation (SD). Statistical analysis was performed using GraphPad Prism software (version 10.1.0; GraphPad Software, San Diego, CA, USA). Multiple comparisons were assessed using one-way ANOVA followed by the Holm–Sidak post hoc test. The Kruskal–Wallis ANOVA on ranks was applied for non-parametric data, followed by Dunn’s correction. A significance level of *p* < 0.05 was considered statistically significant.

## 5. Patents

Andrea Fekete and Adam Vannay. Novel use of Sigma-1 receptor agonist compounds. Patent No. WO2015118365A1. This patent pertains to the use of S1R agonists, such as fluvoxamine, in preventing and/or treating extracellular matrix deposition and related fibrotic conditions.

## Figures and Tables

**Table 1:** Metabolic parameters of control, diabetic (D), and fluvoxamine-treated diabetic (D + FLU) rats at the end of the 7-week experimental period. Values are represented as means ± SD. * *p* < 0.05 vs. Control, ** *p* < 0.01 vs. Control, *** *p* < 0.001 vs. Control (*n* = 6/group).

Metabolic Parameters	Control	Diabetic (D)	D + FLU
Body weight (g)	410 ± 32.3	292 ± 33.7 ***	275 ± 38.3 ***
Non-fasting blood glucose (mmol/L)	12.0 ± 1.74	37.5 ± 7.33 ***	37.7 ± 2.87 ***
Total cholesterol (mmol/L)	2.03 ± 0.49	2.20 ± 0.84	2.34 ± 1.46
Triglycerides (mmol/L)	1.35 ± 0.69	6.96 ± 2.36 ***	9.97 ± 3.64 ***
Serum glutamate–oxaloacetate transaminase (U/L)	134 ± 22.0	543 ± 171 **	508 ± 303 *
Serum glutamate–pyruvate transaminase (U/L)	54.0 ± 13.8	279 ± 85.6 **	322 ± 192 ***

**Table 2:** Serum creatinine, serum blood urea nitrogen (BUN), creatinine clearance, and protein excretion of control, diabetic (D), and fluvoxamine-treated diabetic (D + FLU) rats. Values are represented as means ± SD. ** *p* < 0.01 vs. Control, *** *p* < 0.001 vs. Control, *## p* < 0.01 vs. D (*n* = 6/group).

Renal Parameters	Control	Diabetic (D)	D + FLU
Serum creatinine (μmol/L)	21.6 ± 2.30	40.2 ± 6.02 **	27.0 ± 5.94 ##
BUN (mmol/L)	7.02 ± 0.51	22.2 ± 6.31 **	15.6 ± 3.02 ##
Creatinine clearance (mL/min)	1.25 ± 0.15	0.60 ± 0.12 ***	0.88 ± 0.27
Protein excretion (g/24 h)	2.22 ± 0.88	4.41 ± 0.33 ***	3.78 ± 0.50

**Table 3:** Primer pairs for quantitative RT-qPCR.

Gene	NCBI Ref No.	Primer Pairs	Product Length (bp)
*Col1a1*	NM_053304.1	Forward: 5′ ACTGGATCGACCCTAACCAA 3′ Reverse: 5′ CGCTTCCATACTCGAACTGG 3′	201
*Col3a1*	NM_032085.1	Forward: 5′ ACAACTGATGGTGCTACTGT 3′ Reverse: 5′ GCATCCCAATTCATCTACATTG 3′	177
*EPAS1*	NG_016000.1	Forward: 5′ GACAAGGAGAAGAAAAGGAGTA 3′ Reverse: 5′ GCTCATAGAACACCTCCGTC 3′	100
*EPO*	NG_021471.2	Forward: 5′ GAGCCCAGAAGGAAGCCATC 3′ Reverse: 5′ GTCAGCAGTGATTGTTCGGA 3′	71
*Fn1*	NM_019143.2	Forward: 5′ TGGTCCTAACAAATCTCCTGC 3′ Reverse: 5′ AGTGGACGGTGAATGAGTTG 3′	165
*Havcr1*	NM_173149.2	Forward: 5′ CGCAGAGAAACCCGACTAAG 3′ Reverse: 5′ CAAAGCTCAGAGAGCCCATC 3′	194
*HIF1A*	NG_029606.1	Forward: 5′ CATAAAGTCTGCAACATGGAAGGT 3′ Reverse: 5′ ATTTGATGGGTGAGGAATGGGTT 3′	148
*IL1B*	NG_008851.1	Forward: 5′ CCAATCTTCATTGCTCAAGTGTC 3′ Reverse: 5′ CATTGCCACTGTAATAAGCCATC 3′	88
*IL6*	NG_011640.1	Forward: 5′ CCACTCACCTCTTCAGAACG 3′ Reverse: 5′ TTTTCACCAGGCAAGTCTCC 3′	208
*Lcn2*	NM_130741.1	Forward: 5′ GGGCTGTCCGATGAACTGAA 3′ Reverse: 5′ CATTGGTCGGTGGGAACAGA 3′	98
*NFKB1*	NG_050628.1	Forward: 5′ GCGGCTCATGTTTACAGCTT 3′ Reverse: 5′ CGAATCTGGATGTCATCTTTCTG 3′	213
*RN18S*	NR_003286.4	Forward: 5′ GGCGGCGACGACCCATTC 3′ Reverse: 5′ TGGATGTGGTAGCCGTTTCTCAGG 3′	136
*Rn18S*	NR_046237.1	Forward: 5′ GCGGTCGGCGTCCCCCAACTTCTT 3′ Reverse: 5′ GCGCGTGCAGCCCCGGACATCTA 3′	105
*SLC2A1*	NG_008232.1	Forward: 5′ GATTGGCTCCTTCTCTGTGG 3′ Reverse: 5′ TCAAAGGACTTGCCCAGTTT 3′	129
*TGFB1*	NM_000660.7	Forward: 5′ CGAAGGCGCCCGGGTTATGC 3′ Reverse: 5′ GCGTGCGGCAGCTGTACATTGACT 3′	174
*TLR2*	NG_016229.2	Forward: 5′ TCGGAGTTCTCCCAGTTTCT 3′ Reverse: 5′ GCTTCAACCCACAACTACCA 3′	169
*TLR4*	NG_011475.2	Forward: 5′ CGTGGAGGTGGTTCCTAATA 3′ Reverse: 5′ GCCTCAGGGGATTAAAGCTC 3′	116
*TNF*	NG_007462.1	Forward: 5′ AACGGAGCTGAACAATAGGC 3′ Reverse: 5′ GGGCGATTACAGACACAACT 3′	176
*VEGFA*	NG_008732.1	Forward: 5′ GAGGAGGGCAGAATCATCAC 3′ Reverse: 5′ AGCCCCACAGGGATTTTCTTGTC 3′	341

## Data Availability

The original contributions presented in this study are included in the article. Further inquiries can be directed to the corresponding author.
